# A Novel Heterozygous Mutation in the *STAT1* SH2 Domain Causes Chronic Mucocutaneous Candidiasis, Atypically Diverse Infections, Autoimmunity, and Impaired Cytokine Regulation

**DOI:** 10.3389/fimmu.2017.00274

**Published:** 2017-03-13

**Authors:** Kornvalee Meesilpavikkai, Willem A. Dik, Benjamin Schrijver, Nicole M. A. Nagtzaam, Angelique van Rijswijk, Gertjan J. Driessen, Peter J. van der Spek, P. Martin van Hagen, Virgil A. S. H. Dalm

**Affiliations:** ^1^Department of Immunology, Erasmus University Medical Center, Rotterdam, Netherlands; ^2^Department of Internal Medicine, Division of Clinical Immunology, Erasmus University Medical Center, Rotterdam, Netherlands; ^3^Faculty of Medicine, Department of Microbiology, Chulalongkorn University, Bangkok, Thailand; ^4^Laboratory Medical Immunology, Erasmus University Medical Center, Rotterdam, Netherlands; ^5^Department of Pediatrics, Division of Infectious Disease and Immunology, Erasmus University Medical Center, Rotterdam, Netherlands; ^6^Department of Bioinformatics, Erasmus University Medical Center, Rotterdam, Netherlands

**Keywords:** chronic mucocutaneous candidiasis, signal transducer and activator of transcription 1, Src homology 2 domain, heterozygous mutation, gain-of-function mutation

## Abstract

Chronic mucocutaneous candidiasis (CMC) is a primary immunodeficiency characterized by persistent or recurrent skin and mucosal surface infections with *Candida* species. Different gene mutations leading to CMC have been identified. These include various heterozygous gain-of-function (GOF) mutations in signal transducer and activator of transcription 1 (*STAT1*) that are not only associated with infections but also with autoimmune manifestations. Recently, two *STAT1* GOF mutations involving the Src homology 2 (SH2) domain have been reported, while so far, over 50 mutations have been described mainly in the coiled coil and the DNA-binding domains. Here, we present two members of a Dutch family with a novel *STAT1* mutation located in the SH2 domain. T lymphocytes of these patients revealed STAT1 hyperphosphorylation and higher expression of STAT1 target genes. The clinical picture of CMC in our patients could be explained by diminished production of interleukin (IL)-17 and IL-22, cytokines important in the protection against fungal infections.

## Introduction

**Figure 1:**
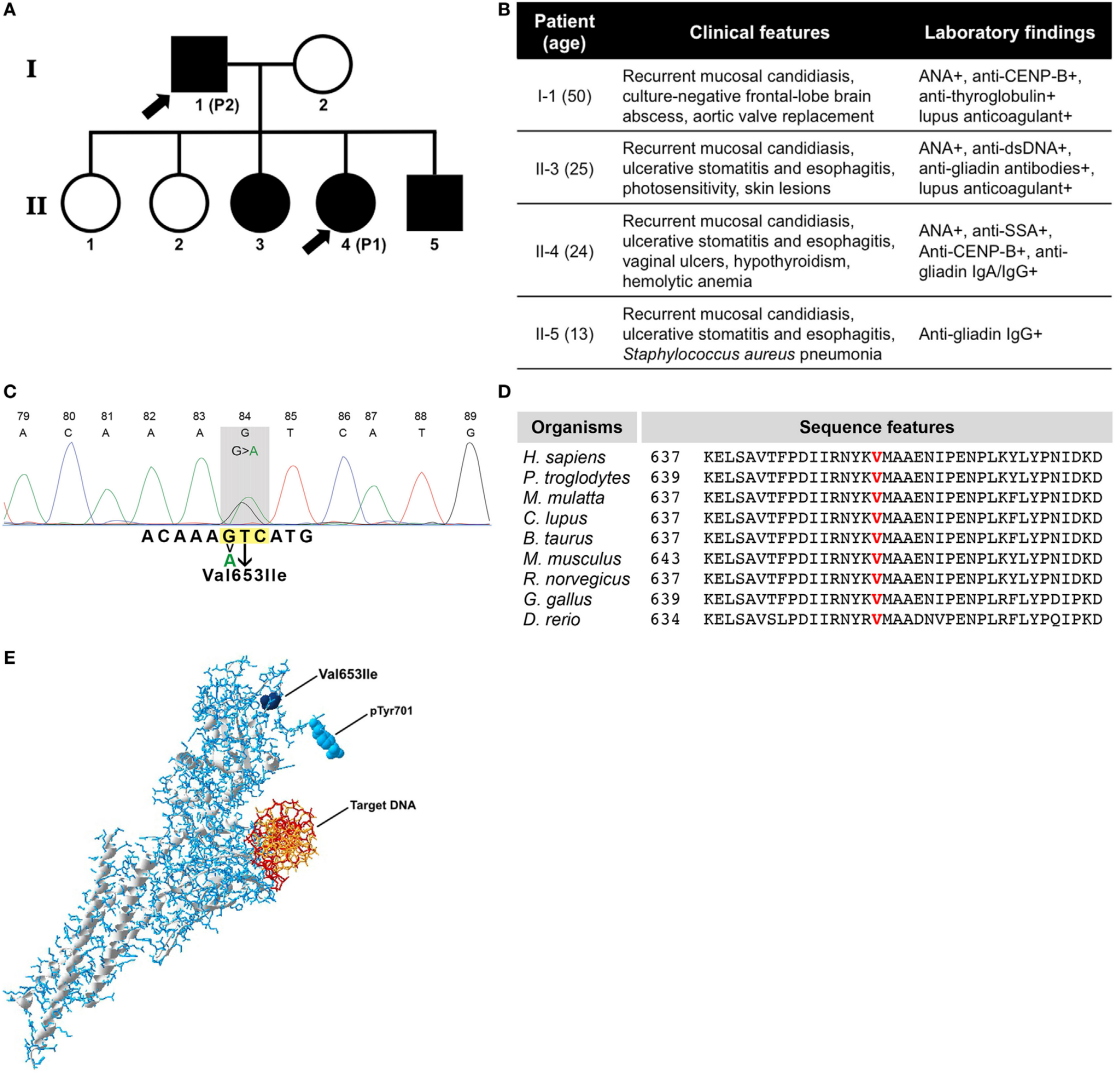
**(A)** Family pedigree of patients. Symbols in black indicate individuals with the same genetic defect and arrow signs indicate the patients enrolled in this study. **(B)** Table showing clinical data of all four affected individuals. **(C)** Sanger DNA sequencing chromatogram of mutated *STAT1* gene. **(D)** Evolutionary conservation of p.Val653 among species. **(E)** Three-dimensional structure of phosphorylated STAT1 protein with the mutation (Val653Ile), the phosphorylation site (pTyr701), and the target DNA indicated.

Patient 1 (II-4) (Figures 1A,B) is a 24-year-old female who presented at age of 1 with a *Streptococcus haemolyticus* jaw abscess, with recurrent oral and esophageal *Candida albicans* infections from the age of 6 onward and an episode of *Staphylococcus aureus* pneumonia and cytomegalovirus (CMV) pneumonia at age of 7 years. At age of 8, she developed hypothyroidism, and there were serologic signs of celiac disease, without clinical relevance. Moreover, additional immunological analysis revealed the presence of antinuclear antibodies (ANA) in high titer (1:5,120), anti-Sjögren’s-syndrome-related antigen A (Ro), and anti-centromere protein B autoantibodies (anti-CENP-B). At the age of 20, she developed autoimmune hemolytic anemia. Over the past years, the clinical picture has been dominated by recurrent oral and esophageal *C. albicans* infections for which she was repetitively treated with antifungal therapies. To prevent recurrence of *C. albicans* infections, she is currently treated with prophylactic antifungal therapy (fluconazole 200 mg once daily). Moreover, she has experienced oral and vaginal ulcers which were found negative for bacterial, fungal, and viral microbes as determined by culture and/or PCR of oral and vaginal swabs and tissue biopsies from vaginal ulcers. Because of these ulcers, which were assumed to be autoimmune manifestations related to chronic mucocutaneous candidiasis (CMC), various immunosuppressive therapies have been initiated over time, including steroids, azathioprine, hydroxychloroquine, and mycophenolate mofetil, all with little benefit. She has recently started treatment with adalimumab [anti-tumor necrosis factor (TNF)-α] 40 mg every other week, which resulted in complete resolution of oral and vaginal ulcers after three subcutaneous injections. After three months of treatment, there is still no recurrence of oral and/or vaginal ulcers, whereas no increased incidence of infectious complications was reported while using anti-TNF-α treatment.

Peripheral blood total CD3+ T lymphocyte numbers [1.63 × 10^9^/l (reference value: 0.7–2.1 × 10^9^/l)], CD4+ T lymphocyte numbers [0.6 × 10^9^/l (reference value: 0.3–1.4 × 10^9^/l)], CD8+ T lymphocyte numbers [0.84 × 10^9^/l (reference value: 0.2–0.9 × 10^9^/l)], total CD19+ B lymphocyte numbers [0.16 × 10^9^/l (reference value: 0.1–0.4 × 10^9^/l)], and CD16+ CD56+ NK cell numbers [0.1 × 10^9^/l (reference value: 0.1–0.4 × 10^9^/l)] were within the normal reference ranges. Immunoglobulin levels were also found to be within normal limits [IgG 10.5 g/l (reference value: 7.0–16.0 g/l), IgA 1.24 g/l (reference value: 0.76–3.91 g/l), and IgM 1.12 g/l (reference value: 0.45–2.30 g/l)]. The patient was negative for autoantibodies against interleukin (IL)-17A, IL-17F, and IL-22. After immunization with a polysaccharide vaccine against *Streptococcus pneumonia* (Pneumovax), IgG antibody concentration to all 16 serotypes (1, 3, 4, 5, 6B, 7F, 8, 9V, 14, 15B, 18C, 19A, 19F, 20, 23F, and 33F) measured increased when compared to pre-vaccination concentrations and for 12 serotypes the post-immunization concentrations reached values above 1 μg/ml, which is indicative of a normal response to polysaccharide immunization (1).

Patient 2 (I-1) (Figures 1A,B) is the 50-year-old father of patient 1, with a medical history including aortic valve replacement at age 29 and surgical and antibiotic treatment for a culture-negative, frontal-lobe brain abscess at age 33. He presented for the first time at our outpatient clinic at the age of 49 because of very severe *C. albicans* infection in the oral cavity and esophagus. At that time, he had already suffered from recurrent oral and esophageal fungal infections for years, for which he was treated with antifungal therapies by his general physician. Microbiological analysis revealed a fluconazole-resistant *C. albicans* and treatment with posaconazole 400 mg once daily was initiated with prompt clinical improvement. Subsequently, prophylactic posaconazole 200 mg once daily has been prescribed. At the time of first analysis, also autoimmune hypothyroidism with positivity for antithyroglobulin antibodies, ANA and lupus anticoagulant were detected. Peripheral blood total CD3+ T lymphocyte numbers [1.06 × 10^9^/l (reference value: 0.7–2.1 × 10^9^/l)], CD4+ T lymphocyte numbers [0.5 × 10^9^/l (reference value: 0.3–1.4 × 10^9^/l)], CD8+ T lymphocyte numbers [0.52 × 10^9^/l (reference value: 0.2–0.9 × 10^9^/l)], total CD19+ B lymphocyte numbers [0.35 × 10^9^/l (reference value: 0.1–0.4 × 10^9^/l)], and CD16+ CD56+ NK cell numbers [0.16 × 10^9^/l (reference value: 0.1–0.4 × 10^9^/l)] were within the normal reference range. Immunoglobulin levels were also found within normal limits [IgG 12.9 g/l (reference value: 7.0–16.0 g/l), IgA 1.65 g/l (reference value: 0.76–3.91 g/l), and IgM 0.8 g/l (reference value: 0.45–2.30 g/l)]. The patient was negative for autoantibodies against IL-17A, IL-17F, and IL-22.

Sanger sequencing from all affected individuals in the family revealed a novel heterozygous *signal transducer and activator of transcription 1 (STAT1)* mutation in exon 22 at c.1957G>A, while genetic testing for mutations in 276 other known genes involved in primary immunodeficiency was negative (Table S1 in Supplementary Material). The nucleotide base change we identified has not been reported as single nucleotide polymorphism (the Human Gene Mutation Database, the National Center of Biotechnology Information, the ExAC database, the 1000G database, and the Ensembl database). The identified mutation results in replacement of a highly conserved valine at position 653 (vertebrate Phylop100 score 3.798 and SiPhy score 19.656) (Figures 1C,D; Table S2 in Supplementary Material) into isoleucine [p.(Val653Ile)] within the Src homology 2 (SH2) domain of STAT1. The mutation is exposed on the outer surface of the molecule and within the vicinity of the phosphorylation site (Figure 1E; Video S1 in Supplementary Material). The Val653Ile mutation is predicted to hardly affect the overall structure of STAT1 and was predicted as “tolerated” by the Sort Intolerant From Tolerant algorithm (score 0.58). However, Val653Ile was predicted as “possibly damaging” by the Polymorphism Phenotyping v2 (PolyPhen-2, score 0.919), the mutation was speculated as “disease causing” from the Mutation Taster and was scaled in Combined Annotation Dependent Depletion algorithm with a score of 14.99, which suggests potential deleteriousness.

**Figure 2:**
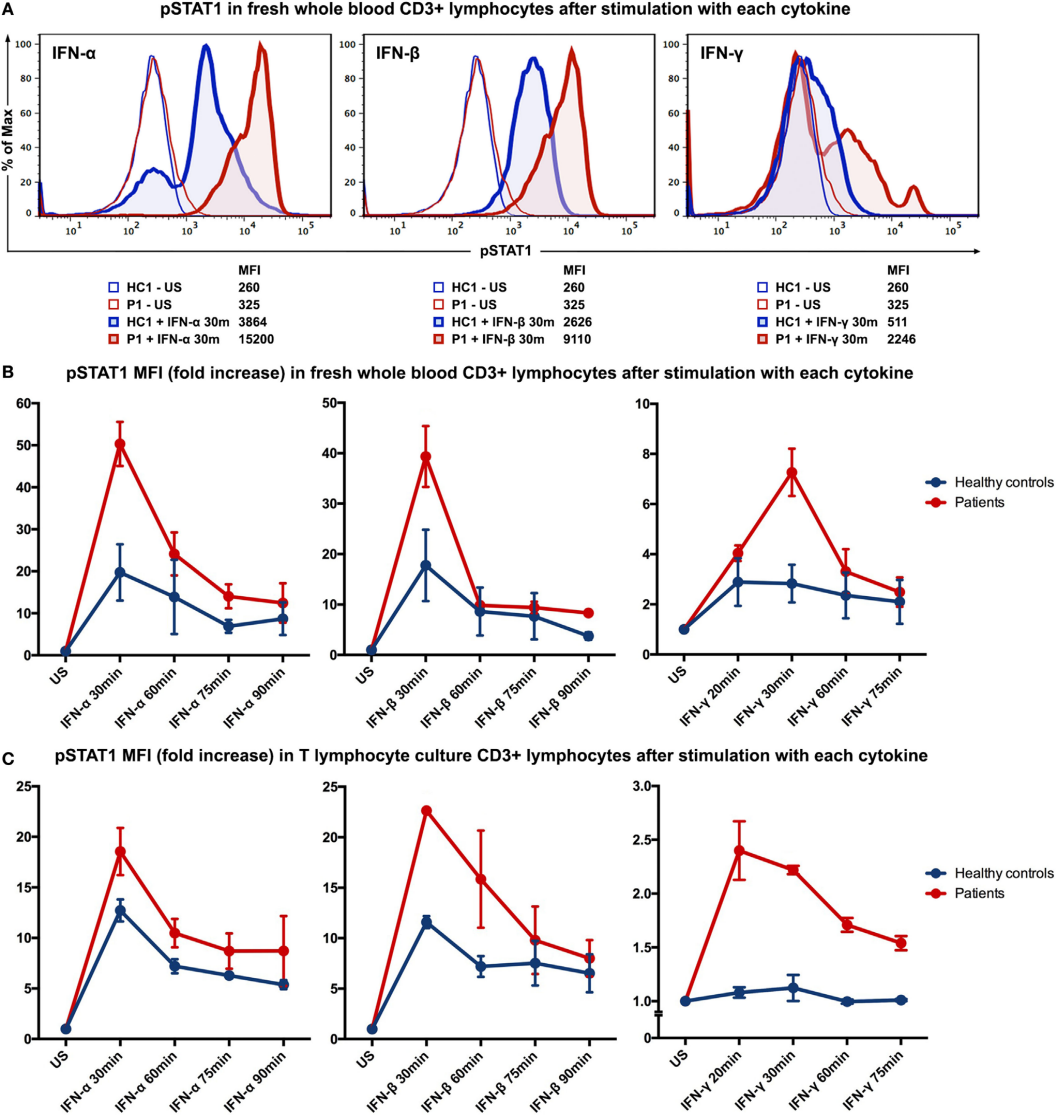
**STAT1 phosphorylation evaluated by intracellular staining flow cytometry after stimulation with IFN-α, IFN-β, or IFN-γ**. **(A)** Histograms showing MFI of phosphorylated STAT1 (pSTAT1) in CD3+ T lymphocytes (in fresh whole blood) of patient P1 and healthy control (HC1) after stimulation with IFN-α, IFN-β or IFN-γ for 30 min. **(B)** Kinetics of STAT1 phosphorylation in CD3+ T lymphocytes (in fresh whole blood) of the two patients and two healthy controls after stimulation with IFN-α, IFN-β, or IFN-γ. **(C)** Kinetics of STAT1 phosphorylation in T lymphocyte cultures from both patients and healthy controls. HC, healthy control; P, patient; US, unstimulated; MFI, mean fluorescence intensity.

To evaluate the immunological phenotype associated with this mutation, STAT1 phosphorylation was studied by flow cytometry of fresh whole blood samples from patient 1 and an age-gender-race-matched healthy control. After stimulation with interferon (IFN)-α (10^4^ IU/ml; PeproTech, London, UK), IFN-β (10^3^ IU/ml; tebu-bio, Le-Perray-en-Yvelines, France), IFN-γ (10^5^ IU/ml; R&D systems, Abingdon, UK) for 30 min, or IL-6 (100 ng/ml; R&D systems) for 15 min, cells were fixed and permeabilized with permeabilizing reagent (Phospho-Epitopes Exposure kit; Beckman Coulter). All samples were stained with CD3 (APC-conjugated antihuman CD3; BD Biosciences, CA, USA) and Fluor^®^ 488-conjugated phospho-STAT1 Tyr701 antibodies (Cell Signaling Technology, MA, USA). The levels of STAT1 phosphorylation in the CD3+ T lymphocytes from the patient were higher than the levels observed in the healthy control after stimulation with IFNs (Figure 2A) or IL-6 (data not shown). To study the kinetics of STAT1 phosphorylation in more detail, time-course stimulation experiments were performed with fresh whole blood samples from patients 1 and 2 along with age-gender-race-matched healthy controls. The patients clearly displayed higher levels of STAT1 phosphorylation, yet the phosphorylation levels of both the patients and controls normalized over time (Figure 2B).

Because we found a higher basal level of phosphorylated STAT1 (pSTAT1) in patient 1 (Figure S1 in Supplementary Material), we established T lymphocyte cultures to reduce the confounding effects of exposure of both patients to different pathogens and therapies. Long-term T lymphocyte cultures were expanded from peripheral blood mononuclear cells (PBMC) of both patients and two age-gender-race-matched healthy controls in RPMI-1640 medium (Lonza, Basel, Switzerland), containing 10% heat inactivated human serum and antibiotics (2% penicillin and streptomycin; Cambrex BioWhittaker, Verviers, Belgium) in the presence of Phytohemagglutinin (1 μg/ml; Sigma-Aldrich, MO, USA), IL-2 (25 IU/ml; Novartis, Basel, Switzerland), IL-15 (12.5 ng/ml; BioLegend, CA, USA), and γ-irradiated (40 Gy) allogeneic PBMC and EBV-negative B lymphocytes. After 2 weeks of culturing, T lymphocyte cultures (purity>90%) from both patients and healthy controls were analyzed for STAT1 phosphorylation in a manner similar to the whole blood samples. IFN stimulation resulted in higher generation of pSTAT1 in the T lymphocyte cultures from patients compared to healthy controls (Figure 2C). Stimulation of T lymphocyte cultures with IL-6 (100 ng/ml) yielded comparable results on pSTAT1 as IFN-γ (data not shown). T lymphocytes from the patients and healthy controls displayed similar levels of total STAT1 protein [determined by flow cytometry using a PE-conjugated STAT1 antibody (BD Biosciences; data not shown)]. STAT3 phosphorylation was also evaluated after stimulation with IL-6 (100 ng/ml) or IL-21 (200 ng/ml; Life Technologies, MA, USA) but did not differ between patients and healthy controls (data not shown).

**Figure 3:**
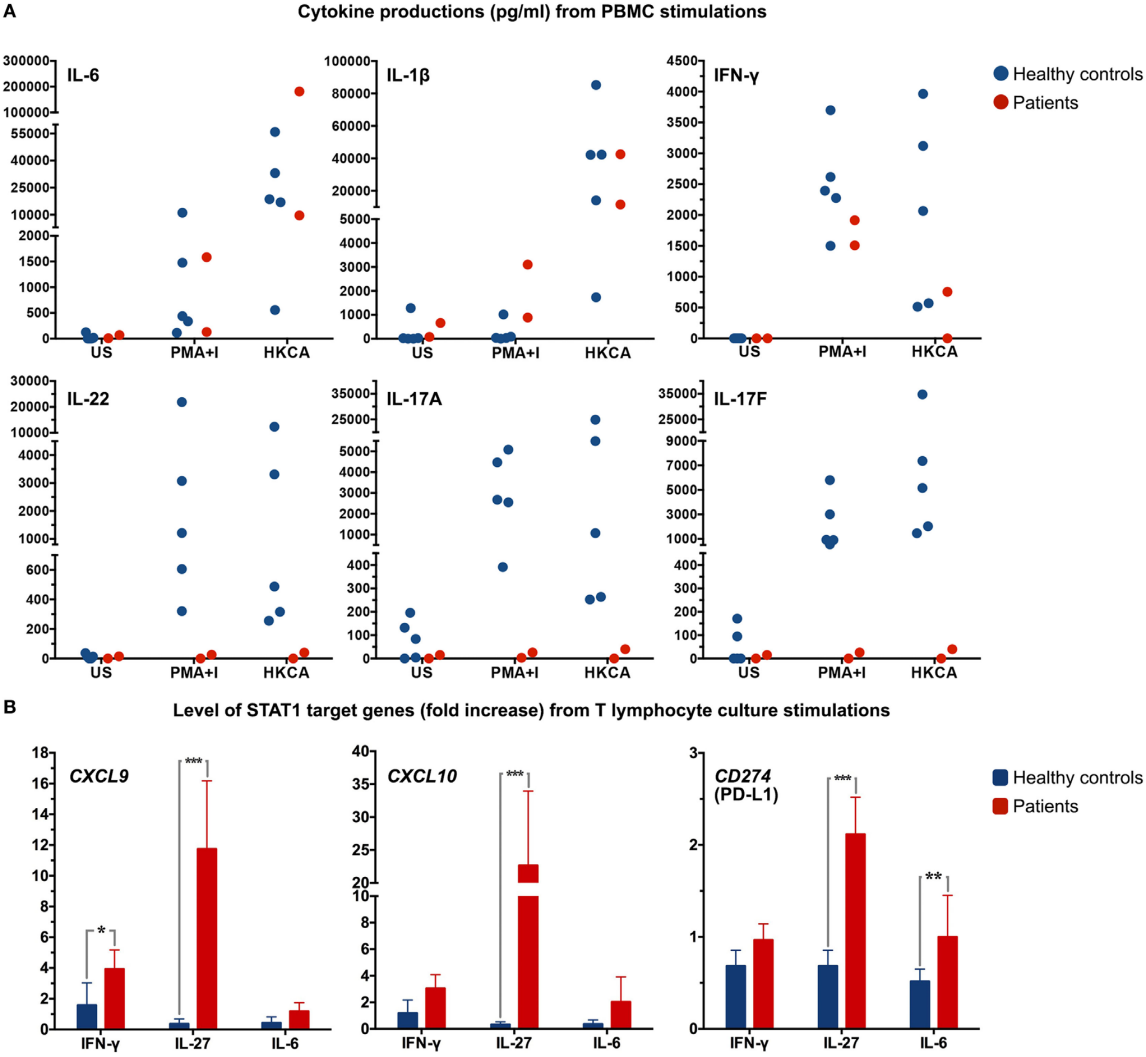
**(A)** Dot plots depicting cytokine production in supernatant from peripheral blood mononuclear cells (PBMC) from both patients and healthy controls (*n* = 5) after stimulation of 10^6^ PBMC with PMA-ionomycin (PMA+I) or heat-killed *C. albicans* (HKCA). Every symbol indicates an individual. US, unstimulated. **(B)** T lymphocyte cultures were stimulated for 24 h with IFN-γ, IL-27, or IL-6 and mRNA expression levels of *CXCL9, CXCL10*, and *CD274* (PD-L1) were determined by real-time quantitative Taqman PCR. Data were normalized to the housekeeping gene, *ABL*. *P < 0.05, **P < 0.01, ***P < 0.001 when compared with healthy controls.

To assess the effect of the *STAT1* mutation on cytokine production, PBMC of both patients and five race-matched healthy controls were freshly prepared and stimulated with heat-killed *C. albicans* [heat-killed *Candida albicans* (HKCA): 10^6^ cells]. Supernatants were collected, and levels of cytokines were measured by enzyme-linked immunosorbent assay (R&D systems). Cells were also stimulated with phorbol 12-myristate 13-acetate (PMA) (81 nM) and ionomycin (I) (1.3 μM; eBioscience, CA, USA) as positive control. PBMC from both patients produced IL-6 and IL-1β levels comparable to that of healthy controls, while PBMC from patient 1 did not produce IFN-γ when stimulated with HKCA (Figure 3A). In contrast to PBMC from healthy controls, PBMC from the patients hardly increased IL-17A, IL-17F, and IL-22 production upon stimulation with PMA-I or HKCA (Figure 3A). STAT1 downstream target genes were also measured in T lymphocyte cultures. Due to limited T lymphocyte numbers in these cultures, only one time-point of stimulation (24 h) with three different stimuli [IFN-γ (10^5^ IU/ml), IL-6 (100 ng/ml), and IL-27 (200 ng/ml; R&D systems)] was examined. *CXCL9, CXCL10*, and *CD274* (PD-L1) mRNA expression levels were determined by real-time quantitative Taqman PCR in each sample on the basis of six replicates. Strikingly high mRNA levels were observed after IL-27 activation (*P* < 0.001) (Figure 3B).

## Background

Chronic and/or recurrent fungal infections may be predominant manifestations in primary immunodeficiencies, especially in inherited T lymphocyte defects. Various syndromes have been identified that present with recurrent fungal infections, for example, autosomal dominant (AD) hyper-immunoglobulin E syndrome, autosomal recessive autoimmune polyendocrine syndrome type I, and Mendelian susceptibility to mycobacterial diseases. AD-CMC is a rare and severe immunodeficiency that presents with severe mucocutaneous fungal infections, autoimmune phenomena, cerebral aneurysms and increased risk of oropharyngeal, and esophageal cancer (2–4). In the last decade, various heterozygous gain-of-function (GOF) mutations in *STAT1* were found to be responsible for AD-CMC. So far, GOF mutations were described in the coiled-coil domain and the DNA-binding domain of STAT1, and in the past year, the GOF mutations c.1885C>T (p.H629Y) and c.1973G>A (p.N658S) involving the SH2 domain were described (5–7). Chronic and/or recurrent mucocutaneous fungal infections with predominantly *C. albicans* are the major infectious complications in patients with *STAT1* GOF mutations and generally arise in infancy or childhood. However, more than half of the patients encounter bacterial infections, with lower respiratory tract infections most frequently observed. Cutaneous viral infections are also described in about one third of patients (6, 8). In addition, many patients with GOF mutations in *STAT1* develop autoimmune manifestations. Autoimmune thyroid disease is reported in 22% of patients. Cerebral aneurysms and cancers are among the most severe complications that are found more frequently and at younger age in these patients when compared to the general population (6, 8).

## Discussion

The mutation identified in our patients is located in the *STAT1* SH2 domain resulting in recurrent mucocutaneous *C. albicans* infections, which is one of the clinical hallmarks of CMC. The other two family members who carry the same STAT1 mutation but were not included in this study also suffered from recurrent mucocutaneous candidiasis (Figure 1B). In addition to fungal infections, patient 1 showed an atypical susceptibility to a wide range of pathogens, as she experienced *S. haemolyticus* jaw abscess, *S. aureus* pneumonia, and CMV pneumonia. Lower respiratory tract bacterial infections can be found in about half of patients with *STAT1* GOF mutations. However, based on previous reports, CMV pneumonia was described in only 1% of patients. Patient 2 also encountered an intracranial abscess for which surgical and antibiotic treatments were required. Although the pathogenic microorganism could not be isolated, invasive deep-seated abscesses are uncommon for patients with *STAT1* GOF mutations (6). Apart from CMC, atypical features of infections including invasive bacterial infections were also described in other patients carrying a *STAT1* GOF mutation in the SH2 domain (5–7). Both patients 1 and 2 enrolled in this study developed a variety of autoimmune phenomena that were displayed in both clinical and laboratory analyses.

In response to various kinds of infections, STAT1 receives signals from the cell surface receptors and is subsequently phosphorylated at Tyr701. The SH2 domain carries the pTyr701-binding site and accordingly plays an important role in forming a firm cross-linkage dimerization between each STAT1 monomer. The dimers then accumulate in the nucleus, inducing transcription of genes (9). The mutation here described within the SH2 domain of STAT1 results in enhancement of STAT1 phosphorylation both upon stimulation with IFN-α/β and IFN-γ, despite the suggestion that the SH2 domain may not necessarily be required for STAT1 activation by IFN-α/β (10). Moreover, this mutation may possibly affect molecule dimerization due to its specific location within the STAT1 molecule.

We performed STAT phosphorylation analysis of both patients by flow cytometry, which revealed enhancement of STAT1 phosphorylation, while total STAT1 protein was equally measured in T lymphocytes of both patients and healthy controls. After stimulation with cytokines, STAT1 phosphorylation in the T lymphocytes of both patients reached higher levels compared to the healthy controls. In contrast to previous reports studying mutations in other STAT1 domains (11–13), we found no clear prolonged STAT1 phosphorylation in the T lymphocytes from the patients. The difference in the mutated domain of *STAT1* could possibly be the cause of different phosphorylation characteristics. Apart from elevated pSTAT1 induction, we also noticed a higher basal pSTAT1 level in peripheral blood T lymphocytes from patient 1. Stimulation of fresh whole blood from patient 2 yielded comparable results, although this patient showed no elevated basal level of pSTAT1 when compared to healthy controls. However, after re-evaluation in established T lymphocyte cultures, the elevated basal pSTAT1 level previously noted in the whole blood analysis of patient 1 disappeared. Due to the fact that patient 2 received prophylactic antifungal therapy while patient 1 did not at the time of analysis, recurrent exposure of patient 1 to *Candida* antigens could have caused the elevated basal STAT1 phosphorylation we observed in her whole blood analysis. Because dysregulation of STAT3 has also been associated with CMC, we evaluated STAT3 phosphorylation as well, but this was comparable between the patients and healthy controls.

Th17-derived cytokines are crucial in fungal defense mechanisms (14, 15), and therefore, we examined the cytokine production capacity of PBMC of both patients as well as healthy controls upon activation with HKCA. A remarkable impairment in IL-17A, IL-17F, and IL-22 production was found in the patients. In order to further examine the consequence of this novel *STAT1* SH2 domain mutation, STAT1 downstream target genes were assessed. T lymphocyte cultures from both patients and healthy controls were stimulated with IFN-γ, IL-6, and IL-27. Significantly higher mRNA levels of STAT1 downstream target genes were found in both patients, especially upon IL-27 activation, including *CD274* (PD-L1). Overexpression of PD-L1 was previously observed in naïve T lymphocytes of patients with *STAT1* GOF mutation, and cytokine-induced PD-L1 expression in T lymphocytes was found to hamper Th17 induction (16, 17). Our data suggest that disturbed Th17 differentiation and associated cytokine production most likely underlies the clinical picture of CMC in the patients described here.

Since curative treatment is still unavailable, most of the patients with *STAT1* GOF mutations receive prolonged systemic antimicrobial medications to control clinical symptoms of recurrent fungal infections and other infections. Similar to patient 2, about 40% of the patients who require long-term antifungal treatment, eventually develop therapy resistance (6). Immunotherapies or immunosuppressive therapies are considered in some patients, although the effectiveness still needs to be evaluated in more detail (18). Surprisingly, novel therapies, like JAK1/2 inhibitors, showed not only the potency to suppress the enhanced STAT1 phosphorylation in CD4+ T lymphocytes but may also improve clinical outcome in both immunodeficiency and autoimmunity features (13, 19). These drugs could be potential future candidates for the treatment of patients with CMC with *STAT1* GOF mutations.

## Concluding Remarks

The novel Val653Ile mutation, located in the SH2 domain of STAT1 found in this family does not clearly result in an impaired STAT1 dephosphorylation rate, as was found in patients with GOF mutations in the other domains. However, significantly enhanced STAT1 phosphorylation in these patients results in higher expression of the STAT1 target genes *CXCL9, CXCL10*, and *CD274* (PD-L1). Moreover, this mutation is associated with the impairment of immune cells to produce IL-17A, IL-17F, and IL-22. The clinical symptoms of CMC could, therefore, be explained by diminished Th17 responses that are crucial for confronting fungal antigens.

## Ethics Statement

This study was approved by the Medical Ethics Committees of Erasmus MC with ethics approval number MEC 2013-026. Clinical data and blood samples of patients with novel STAT1 mutation and healthy controls were collected after written informed consent was obtained.

## Author Contributions

KM, WD, PH, and VD designed the research. KM, BS, NN, and AR performed the experiments. KM, WD, PS, and VD analyzed the results. KM, WD, GD, PS, PH, and VD wrote the article.

## Conflict of Interest Statement

The authors declare that the research was conducted in the absence of any commercial or financial relationships that could be construed as a potential conflict of interest. The reviewer RS and handling editor declared their shared affiliation, and the handling editor states that the process nevertheless met the standards of a fair and objective review.

## Abbreviations

CMC, chronic mucocutaneous candidiasis; anti-dsDNA, anti-double stranded DNA; STAT1, signal transducer and activator of transcription 1; SH2, Src homology 2; CMV, cytomegalovirus; ANA, antinuclear antibodies; SSA, Sjögren’s-syndrome-related antigen A (Ro); CENP-B, centromere protein B; MFI, mean fluorescence intensity; PBMC, peripheral blood mononuclear cells; ELISA, enzyme-linked immunosorbent assay; HKCA, heat-killed *Candida albicans*; PMA, phorbol 12-myristate 13-acetate; I, ionomycin; qRT-PCR, quantitative reverse transcription polymerase chain reaction.
