# Supplementary material for: A Novel Heterozygous Mutation in the STAT1 SH2 Domain Causes Chronic Mucocutaneous Candidiasis, Atypically Diverse Infections, Autoimmunity, and Impaired Cytokine Regulation
Source: Front Immunol. 2017 Mar 13;8:274. doi: 10.3389/fimmu.2017.00274 (PMC5346540; doi:10.3389/fimmu.2017.00274)
Supplement: Supplementary file 1 [file Data_Sheet_1.DOCX]

**Supplementary Material**

**Table S1**

| List of PID causing genes evaluated in the affected individuals | | | | | |
| --- | --- | --- | --- | --- | --- |
| *ACP5*  *ACTB*  *ADA*  *ADAR*  *AICDA*  *AIRE*  *AK2*  *AP3B1*  *APOL1*  *ATM*  *B2M*  *BCL10*  *BLM*  *BLNK*  *BTK*  *C1QA*  *C1QB*  *C1QC*  *C1R*  *C1S*  *C2*  *C3*  *C4A*  *C4B*  *C5*  *C6*  *C7*  *C8A*  *C8B*  *C8G*  *C9*  *C9orf142*  *CARD11*  *CARD14*  *CARD9*  *CASP10*  *CASP8*  *CCBE1*  *CD16*  *CD19*  *CD20*  *CD21*  *CD247*  *CD27*  *CD3D*  *CD3E*  *CD3G*  *CD3Z*  *CD40*  *CD40LG*  *CD46* | *CD59*  *CD79A*  *CD79B*  *CD81*  *CD8A*  *CEBPE*  *CECR1*  *CFB*  *CFD*  *CFH*  *CFHR1*  *CFHR2*  *CFHR3*  *CFHR4*  *CFHR5*  *CFI*  *CFP*  *CHD7*  *CIITA*  *CLPB*  *COH1*  *COLEC11*  *COPA*  *CORO1A*  *CR2*  *CR3*  *CSF2RA*  *CSF3R*  *CTPS1*  *CTSC*  *CXCR4*  *CYBA*  *CYBB*  *DCLRE1B*  *DCLRE1C*  *DKC1*  *DNMT3B*  *DOCK8*  *DPKC*  *ELANE*  *EPG5*  *FADD*  *FAS*  *FASLG*  *FCN3*  *FERMT3*  *FOXN1*  *FOXP3*  *FPR1*  *G6PC3*  *G6PT1* | *GATA2*  *GFI1*  *HAX1*  *ICOS*  *IFIH1*  *IFNGR1*  *IFNGR2*  *IGLL1*  *IKBKB*  *IKBKG*  *IKZF1*  *IL10*  *IL10RA*  *IL10RB*  *IL12B*  *IL12RB1*  *IL17F*  *IL17RA*  *IL17RC*  *IL1RN*  *IL21R*  *IL2RA*  *IL2RG*  *IL36RN*  *IL7R*  *INO80*  *IRAK4*  *IRF7*  *IRF8*  *ISG15*  *ITCH*  *ITGB2*  *ITK*  *JAGN1*  *JAK3*  *KINDLIN-3*  *KRAS*  *LAMTOR2*  *LCK*  *LIG4*  *LPIN2*  *LRBA*  *LYST*  *MAGT1*  *MALT1*  *MAP3K14*  *MASP1*  *MASP2*  *MCM4*  *MEFV*  *MRE11A* | *MS4A1*  *MSH6*  *MTHFD1*  *MVK*  *MYD88*  *NBN*  *NCF1*  *NCF2*  *NCF4*  *NFAT5*  *NFKB2*  *NFKBIA*  *NHEJ1*  *NHP2*  *NLRP12*  *NLRP3*  *NOD2*  *NOP10*  *NRAS*  *ORAI1*  *OX40*  *PARN*  *PGM3*  *PIK3CD*  *PIK3R1*  *PLCG2*  *PLDN*  *PMS2*  *PNP*  *POLE*  *PRF1*  *PRKCD*  *PRKDC*  *PSMB8*  *PSTPIP1*  *PTPRC*  *RAB27A*  *RAC2*  *RAG1*  *RAG2*  *RBCK1*  *RFX5*  *RFXANK*  *RFXAP*  *RHOH*  *RMRP*  *RNASEH2A*  *RNASEH2B*  *RNASEH2C*  *RNF168*  *RNF31* | *RORC*  *RPSA*  *RTEL1*  *SAMHD1*  *SBDS*  *SEMA3E*  *SERPING1*  *SH2D1A*  *SH3BP2*  *SLC29A3*  *SLC35C1*  *SLC37A4*  *SLC46A1*  *SMARCAL1*  *SP110*  *SPINK5*  *STAT1*  *STAT2*  *STAT3*  *STAT5B*  *STIM1*  *STK4*  *STX11*  *STXBP2*  *TAP1*  *TAP2*  *TAPBP*  *TAZ*  *TBK1*  *TBX1*  *TCF3*  *TCN2*  *TERC*  *TERT*  *THBD*  *TICAM1*  *TINF2*  *TLR3*  *TMC6*  *TMC8*  *TMEM173*  *TNFRSF13B*  *TNFRSF13C*  *TNFRSF1A*  *TNFRSF4*  *TNFRSF6*  *TNFSF12*  *TNFSF6*  *TPP1*  *TPP2*  *TRAC* | *TRAF3*  *TRAF3IP2*  *TREX1*  *TRNT1A*  *TTC7A*  *TYK2*  *UNC119*  *UNC13D*  *UNC93B1*  *UNG*  *USB1*  *VPS13B*  *VPS45*  *WAS*  *WIPF1*  *XIAP*  *XLF*  *XRCC4*  *XRCC5*  *XRCC6*  *ZAP70*  *ZBTB24* |

**Table S2**

| **Orthologous amino acid conservation** | | | |
| --- | --- | --- | --- |
| **Taxonomy** | **Protein ID** | **Alignment** | **Similarity** |
| *Homo sapiens* | NP_009330.1 | 644 FPDIIRNYK**V**MAAENIPENPL 664 | 100% |
| *Alligator mississippiensis* | XP_006272431.1 | 644 FPDIIRNYK**V**MAAENIPENPL 664 | 100% |
| *Alligator sinensis* | XP_006035043.1 | 652 FPDIIRNYK**V**MAAENIPENPL 672 | 100% |
| *Anas platyrhynchos* | XP_005011477.1 | 064 FPDIIRNYK**V**MAAENIPENPL 084 | 100% |
| *Bos taurus* | XP_005202627.2 | 644 FPDIIRNYK**V**MAAENIPENPL 664 | 100% |
| *Callithrix jacchus* | XP_002749614.1 | 644 FPDIIRNYK**V**MAAENIPENPL 664 | 100% |
| *Cavia porcellus* | XP_003478825.2 | 644 FPDIIRNYK**V**MAAENIPENPL 664 | 100% |
| *Chinchilla lanigera* | XP_005373353.1 | 644 FPDIIRNYK**V**MAAENIPENPL 664 | 100% |
| *Chrysochloris asiatica* | XP_006864113.1 | 644 FPDIIRNYK**V**MAAENIPENPL 664 | 100% |
| *Condylura cristata* | XP_004674489.1 | 644 FPDIIRNYK**V**MAAENIPENPL 664 | 100% |
| *Cricetulus griseus* | XP_003511669.1 | 644 FPDIIRNYK**V**MAAENIPENPL 664 | 100% |
| *Echinops telfairi* | XP_004701556.1 | 644 FPDIIRNYK**V**MAAENIPENPL 664 | 100% |
| *Erinaceus europaeus* | XP_007521470.1 | 487 FPDIIRNYK**V**MAAENIPENPL 507 | 100% |
| *Falco cherrug* | XP_005440520.1 | 648 FPDIIRNYK**V**MAAENIPENPL 668 | 100% |
| *Falco peregrinus* | XP_005241371.1 | 648 FPDIIRNYK**V**MAAENIPENPL 668 | 100% |
| *Gallus gallus* | NP_001012932.1 | 646 FPDIIRNYK**V**MAAENIPENPL 666 | 100% |
| *Gorilla gorilla* | XP_004033014.1 | 644 FPDIIRNYK**V**MAAENIPENPL 664 | 100% |
| *Heterocephalus glaber* | XP_004869016.1 | 644 FPDIIRNYK**V**MAAENIPENPL 664 | 100% |
| *Ictidomys tridecemlineatus* | XP_005324423.1 | 644 FPDIIRNYK**V**MAAENIPENPL 664 | 100% |
| *Jaculus jaculus* | XP_004660304.1 | 644 FPDIIRNYK**V**MAAENIPENPL 664 | 100% |
| *Macaca mulatta* | NP_001248543.1 | 644 FPDIIRNYK**V**MAAENIPENPL 664 | 100% |
| *Monodelphis domestica* | XP_007494594.1 | 643 FPDIIRNYK**V**MAAENIPENPL 663 | 100% |
| *Mus musculus* | NP_001192242.1 | 650 FPDIIRNYK**V**MAAENIPENPL 670 | 100% |
| *Nomascus leucogenys* | XP_004092047.1 | 644 FPDIIRNYK**V**MAAENIPENPL 664 | 100% |
| *Ochotona princeps* | XP_004577343.1 | 591 FPDIIRNYK**V**MAAENIPENPL 611 | 100% |
| *Octodon degus* | XP_004628673.1 | 644 FPDIIRNYK**V**MAAENIPENPL 664 | 100% |
| *Oryctolagus cuniculus* | XP_002712392.2 | 644 FPDIIRNYK**V**MAAENIPENPL 664 | 100% |
| *Ovis aries* | NP_001159675.1 | 644 FPDIIRNYK**V**MAAENIPENPL 664 | 100% |
| *Pan paniscus* | XP_003825332.1 | 644 FPDIIRNYK**V**MAAENIPENPL 664 | 100% |
| *Pan troglodytes* | XP_009442166.1 | 644 FPDIIRNYK**V**MAAENIPENPL 664 | 100% |
| *Papio anubis* | XP_003907787.1 | 644 FPDIIRNYK**V**MAAENIPENPL 664 | 100% |
| *Poecilia formosa* | XP_007541472.1 | 623 LPDIIRTYK**V**MAVENIPENPL 643 | 85% |
| *Pongo abelii* | NP_001126683.1 | 644 FPDIIRNYK**V**MAAENIPENPL 664 | 100% |
| *Python bivittatus* | XP_007420448.1 | 649 LPDIIRSYK**V**MAAENIPENPL 669 | 90% |
| *Rattus norvegicus* | NP_116001.2 | 644 FPDIIRNYK**V**MAAENIPENPL 664 | 100% |
| *Saimiri boliviensis* | XP_003940862.1 | 644 FPDIIRNYK**V**MAAENIPENPL 664 | 100% |
| *Sorex araneus* | XP_004601285.1 | 644 FPDIIRNYK**V**MAAENIPENPL 664 | 100% |
| *Sus scrofa* | NP_998934.1 | 644 FPDIIRNYK**V**MAAENIPENPL 664 | 100% |
| *Tupaia chinensis* | XP_006147877.1 | 644 FPDIIRNYK**V**MAAENIPENPL 664 | 100% |
| *Xenopus laevis* | NP_001082256.1 | 643 FPDIIRNYK**V**MAAENIPENPL 663 | 100% |

**Figure S1**

**
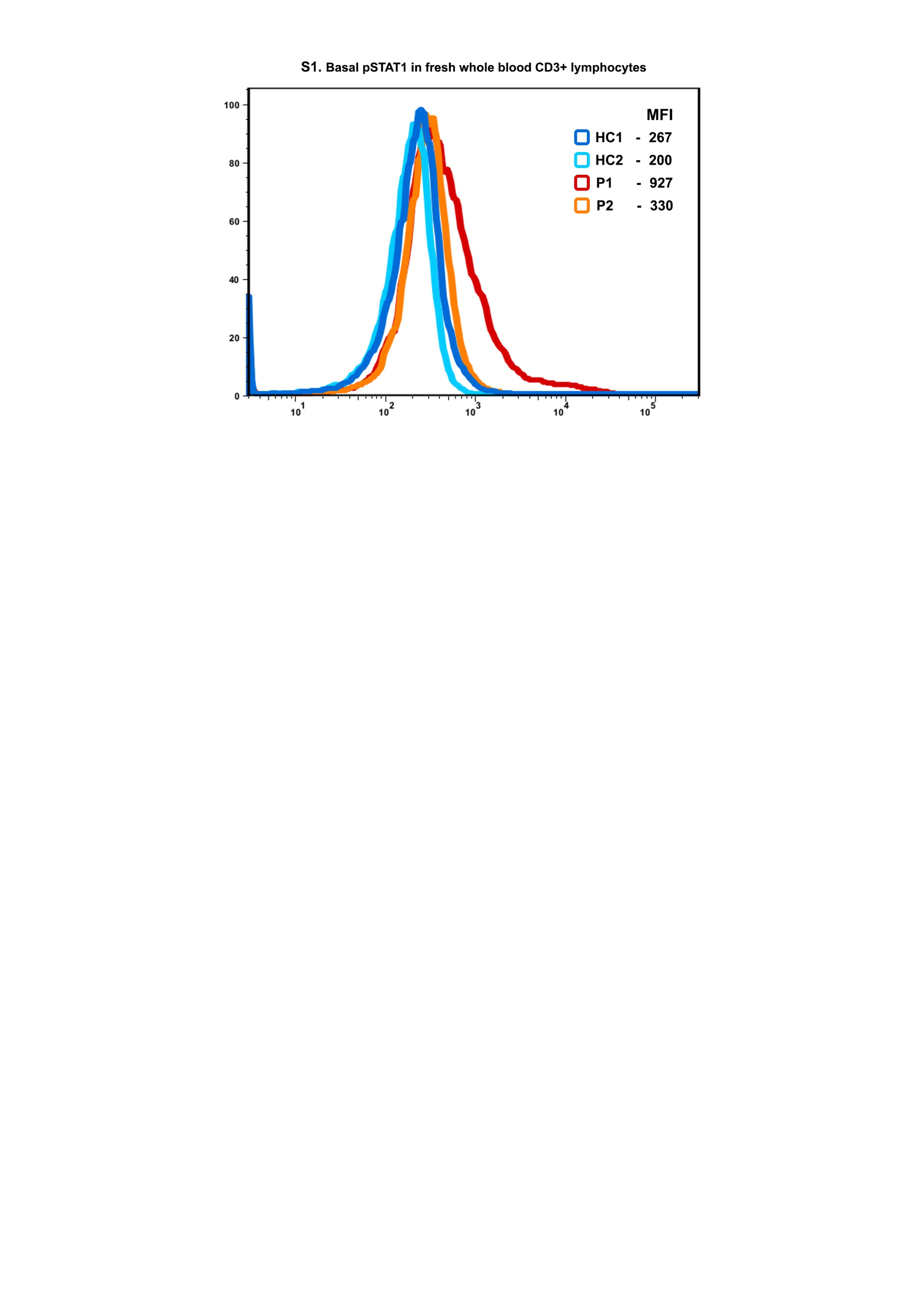
**

**Figure S1:** Histogram showing MFI of phosphorylated STAT1 in CD3+ T lymphocytes evaluated by intracellular staining flow cytometry (in fresh whole blood) at basal level. HC, healthy control; P, patient; MFI, mean fluorescence intensity.
